# Evaluating the Impact on Pain Perceptions, Pain Intensity, and Physical Activity of a Mobile App to Empower Employees With Musculoskeletal Pain: Mixed Methods Pilot Study

**DOI:** 10.2196/67886

**Published:** 2025-06-27

**Authors:** Stijn Keyaerts, Maxwell Szymanski, Lode Godderis, Vero Vanden Abeele, Liesbeth Daenen

**Affiliations:** 1 Knowledge, Information and Research Center Group IDEWE Leuven Belgium; 2 Department of Public Health and Primary Care KU Leuven Leuven Belgium; 3 Department of Computer Science KU Leuven Leuven Belgium; 4 Department of Physiotherapy, Human Physiology and Anatomy VU Brussels Brussels Belgium

**Keywords:** mobile health, mHealth, occupational health, musculoskeletal disorders, mixed methods, pain perceptions, pain management, digital health, human-centered design

## Abstract

**Background:**

Mobile apps present opportunities to empower employees with musculoskeletal pain and reduce long-term absenteeism. However, adoption remains limited because of a lack of empirical evidence and challenges in user-friendly design.

**Objective:**

This pilot study aimed to evaluate the potential effects of a fully automated, app-based pain management intervention tailored for employees. Specifically, the study aimed to (1) assess the effect of the intervention on maladaptive pain perceptions, pain intensity, and physical activity and (2) identify factors influencing its effectiveness.

**Methods:**

A total of 66 employees from a Belgian university hospital who had been experiencing musculoskeletal pain for at least 6 weeks participated in a 24-week intervention. The app-based intervention focused on reducing maladaptive pain perceptions, providing work-related guidance, and promoting healthy activity habits through interactive modules, real-time recommendations, and goal-setting features. Every 6 weeks, participants completed a questionnaire measuring maladaptive pain perceptions (pain catastrophizing and fear-avoidance beliefs). Pain intensity was recorded daily using a visual analog scale, and step count was tracked daily using an activity tracker. In addition, semistructured interviews were conducted with 12 participants to explore how they engaged with the intervention and perceived its impact.

**Results:**

Quantitative analysis showed a significant reduction in pain catastrophizing (B=−0.83, *P*<.001, *d*=−0.27), with greater decreases observed among participants with higher baseline scores (σ=−0.38; *P*=.09). No significant overall change was found in fear-avoidance beliefs (B=−0.35; *P*=.15), although individual trajectories varied (σ²=1.34; *P*=.04). Pain intensity also showed significant variability across participants (σ²=17.29; *P*=.03) despite no overall effect (B=−0.37; *P*=.67). No significant change was observed in the daily step count (B=107.50; *P*=.23). Qualitative analysis revealed that the effectiveness of the intervention was hindered by content and design choices that did not adequately account for diverse work settings and the busy lives of employees. Cognitive biases and nonsupportive work environments further complicated the successful implementation of the intervention in the workplace.

**Conclusions:**

This pilot study demonstrates the potential of an app-based intervention to support employees with musculoskeletal pain by reducing pain-related fear and promoting active coping strategies. While promising for some, digital interventions alone may be insufficient for employees with more complex needs. Blended approaches and integration within supportive workplace environments are likely essential to enhance effectiveness and promote sustainable work participation.

## Introduction

### Background

The European Agency for Safety and Health at Work has highlighted that 58% of the European working population is affected by musculoskeletal pain, which is significantly higher than the global average of 34% [1,2]. Musculoskeletal conditions are a major cause of long-term absenteeism, responsible for up to 60% of cases in Europe [3]. In Belgium, these concerns accounted for 32% of long-term sickness absence in 2022, second only to mental health conditions [4]. These statistics underscore the urgent need for effective interventions to reduce the burden of musculoskeletal pain on employees and the workforce.

However, despite the pressing need, access to appropriate interventions remains a substantial challenge. European legislation mandates periodic health examinations based on occupational risk exposure, leaving approximately 30% of employees without proactive occupational health support [5,6]. Furthermore, the growing shortage of occupational physicians, combined with the limited capacity of specialized pain centers, exacerbates delays in care [7-9]. In Belgium, waiting times for treatment at a specialized center range from 1 to 3 months [10]. By the time employees receive treatment, many have already experienced prolonged sickness absence, making return to work more difficult [11]. These barriers highlight the urgent need for scalable workplace health strategies that facilitate timely interventions to prevent long-term absenteeism.

Mobile health (mHealth) technologies use mobile devices to provide information, deliver treatments, and support self-monitoring, allowing individuals to manage their conditions more conveniently [12,13]. The growing popularity of mHealth has led to the rapid expansion of pain management apps, with 279 apps already available by 2014 [14]. However, most of these apps have substantial limitations. Nearly 60% provide only basic self-care information, lacking essential behavioral components such as goal setting. Moreover, 92% of these apps were developed without input from end users or domain experts, and only 0.4% had undergone clinical validation, raising concerns about their effectiveness.

More recently, efforts to translate validated pain management interventions (eg, mindfulness, education, and guided exercise) into digital formats have shown promising results [15-18]. However, these pain management apps have not been designed to address the unique challenges of employees managing musculoskeletal pain in the workplace. A systematic review by Grant et al [19] identified symptom management, work relations, and workplace adjustments as major barriers to returning to work for employees with musculoskeletal pain. In addition, pain-related fear was highlighted as a major challenge. According to the fear-avoidance model, maladaptive pain perceptions (eg, pain catastrophizing and fear-avoidance beliefs) contribute to hypervigilance and activity avoidance, increasing the risk of persistent pain and disability [20-24]. Research further suggests that pain perceptions and work-related factors interact, influencing the risk of pain and related absenteeism [25,26]. Notably, Cullen et al [27] found that return-to-work interventions that integrate health improvement, work relations, and modified working conditions are more effective than single domain approaches. Therefore, mHealth interventions for employees with musculoskeletal pain should integrate evidence-based pain management strategies within the broader occupational context.

### Objectives

To address this gap, we developed an app-based intervention to provide actionable pain management strategies within the occupational context. The intervention focused on reducing maladaptive pain perceptions, providing work-related guidance, and promoting healthy activity habits through interactive modules, real-time recommendations, and goal-setting features. The app was developed using a human-centered design approach, involving 320 employees with musculoskeletal pain and 8 health care professionals [28,29]. This iterative process ensured that the final prototype was user-friendly and aligned with employees’ needs.

Establishing the clinical effectiveness of this intervention is essential for its integration into workplace health strategies. This pilot study evaluated its impact on maladaptive pain perceptions. Given the role of maladaptive pain perceptions in pain persistence and avoidance behavior, this pilot study also assessed whether the intervention contributed to reductions in pain intensity and improvements in physical activity [20]. Finally, factors influencing the intervention’s effectiveness were explored, as understanding these factors is critical for optimizing the intervention in the future [30].

## Methods

### Study Design

Participants were recruited between August and October 2021 using a convenience sampling approach. Employees from a Belgian university hospital were informed about the project through various channels, including mailings and brochures. Those interested were asked to contact the researchers for additional information. Eligible participants were aged at least 18 years, had been experiencing musculoskeletal pain for at least 6 weeks, were proficient in Dutch, had access to a smartphone (Android or iOS), and provided written or electronic informed consent. Exclusion criteria included pregnancy or planned retirement between November 2021 and May 2022.

In November 2021, the participants received a Fitbit Inspire activity tracker (Fitbit Inc) and were granted access to the pain management app, Health Empower (available on the App Store and Play Store). Upon logging in and securing their accounts, participants were asked to complete demographic and background information along with the baseline questionnaire. They were encouraged to use the Health Empower app and Fitbit for 24 weeks and complete a diary and 6-weekly follow-up questionnaires.

After 24 weeks, participants were invited to participate in a semistructured interview. These web-based interviews, conducted via Microsoft Team, lasted approximately 60 minutes. At the start of the interviews, participants were briefed on the procedure and purpose. The interviews were recorded, transcribed verbatim, and the recordings were subsequently deleted.

### Ethical Considerations

This pilot study was approved by the Ethics Committee Research UZ/KU Leuven (S-65610) and adhered to Belgian and international privacy and ethical regulations. All participants provided written informed consent before participation. The consent process ensured that participants were fully informed about the study’s purpose, procedures, potential risks, and benefits before agreeing to participate.

Participation in the study was voluntary, and participants could withdraw at any time without any consequences. There was no cost or fee associated with participation, and no financial compensation was offered.

To maintain participant confidentiality, all collected data were stored in secure, access-controlled databases and were deidentified before analysis. All data presented in this manuscript and in any supplementary materials are fully anonymous and cannot be traced back to individual participants.

### Intervention

**Figure 1:**
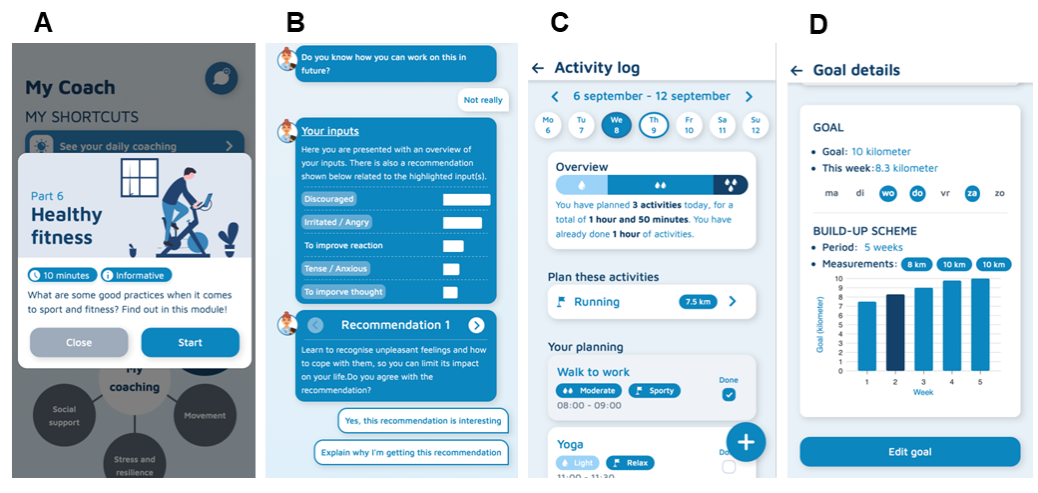
Example screens of the Health Empower app: (A) coaching modules; (B) pain reaction record; (C) activity log; and (D) goal setting.

The app-based intervention consisted of 6 modules, each addressing distinct aspects of pain management through information, exercises, and actionable recommendations (Figure 1). Modules 1 and 2 focused on improving cognitive and affective responses to pain. Module 1 included pain neuroscience education, explaining the neurophysiology of pain and its contributing factors, aiming to reconceptualize pain and reduce its threat value [31,32]. Participants had to complete this module before gaining access to the other modules. The second module expanded on recognizing and addressing unhelpful thoughts and emotions through cognitive behavioral therapy, including mindfulness techniques [33]. Modules 3, 4, and 5 focused on the work context. In the third module, participants learned about ergonomics and activity management [34,35]. The information was tailored to sedentary and physically demanding jobs, including guidelines for workstation organization, manual handling of loads, work postures, work-rest cycles, and activity pacing. Ergonomic advice was aligned with pain neuroscience education to avoid conflicting messages [31,36,37]. The fourth module focused on stress management and resilience training, including self-regulation, problem-solving, and goal-setting techniques [33,38,39]. The fifth module promoted social support through empathic communication and conflict resolution skills [38]. The final module encouraged healthy lifestyle habits, offering advice on physical activity, sleep, and nutrition [40,41].

The app also featured a pain reaction record and an activity log (Figure 1) [31,42]. The pain reaction record allowed users to select statements reflecting their thoughts and coping strategies during pain flare-ups, with tailored recommendations provided by a hierarchical algorithm to improve their response in future situations. The development of the pain reaction record and recommender system is discussed elsewhere [43,44]. The activity log included tools for planning activities, setting personal goals, and monitoring progress [45,46]. To support goal setting and behavioral change, the app was integrated with the Fitbit Inspire activity tracker, allowing participants to synchronize their daily step count. If a participant set a daily step goal in the app, Fitbit data were used to track progress within their graded activity scheme, helping them gradually increase physical activity in a structured manner.

To support sustained engagement with the app and study procedures, several built-in features were included. Participants were limited to completing 1 new module chapter per day, encouraging spaced learning and promoting daily app interaction. All previously completed content remained accessible for review at any time. To enhance adherence to the study protocol, a visual timer was displayed for both the daily (24-h window) and 6-weekly (7-d window) questionnaires, indicating how much time remained for completion. Participants could also enable push notifications to receive alerts when a new coaching chapter or questionnaire became available. In addition, they had the option to set up custom notifications at preferred times and days of the week. These reminders were triggered if a participant had not yet accessed the coaching content that day or if there was still an open questionnaire requiring completion.

### Measurements

#### Sociodemographic and Background Information

Musculoskeletal pain concerns were assessed using the Dutch Musculoskeletal Questionnaire, where participants reported pain or discomfort in specific body regions over the past week (ie, the neck, shoulders, back, elbows, wrists and hands, hips, knees, or ankles and feet) [47]. Multisite pain was considered if participants reported pain in more than 1 body part. Participants also provided information on the duration of concerns and the frequency of absenteeism because of pain in the previous year. Additional sociodemographic variables collected included age, sex, level of education, profession, work arrangement, and tenure.

#### Work-Related Factors

Work-related factors measured at baseline included physical job demands, workload, job autonomy, and social support. Physical job demands were evaluated using 4 of 12 items from the Dutch Musculoskeletal Questionnaire [47]. Participants rated the frequency of engaging in various physical tasks, such as standing, sitting, or handling loads, on a scale from (almost) never to (almost) always. Exploratory factor analysis identified 2 factors with eigenvalues >1.00 (Multimedia Appendix 1). One factor, representing physically demanding work, included 4 items and demonstrated good internal consistency (Cronbach α=0.86). The composite score for this factor ranged from 4 to 16, with higher scores indicating greater physical demands at work. Workload and social support were evaluated using subscales of the Short Inventory to Monitor Psychosocial Hazards, a validated questionnaire that assesses major psychosocial hazards at work [48]. The workload subscale, consisting of 3 items (scores ranging from 3 to 15), measures the perceived volume of tasks and associated time pressures. The social support subscale, consisting of 4 items (scores ranging from 4 to 20), measures the perceived availability and appreciation from colleagues and supervisors. Both the workload (Cronbach α=0.83) and social support (Cronbach α=0.76) subscales showed good reliability, with higher scores indicating higher workload and greater social support. Job autonomy was measured using a subscale of the Questionnaire on the Experience and Evaluation of Work (version 2.0), a validated questionnaire measuring psychosocial well-being and work-related stress [49]. The autonomy subscale, consisting of 4 items (scores ranging from 4 to 20), evaluates the degree of control and discretion individuals have over various aspects of their work environment, including task planning and choice of work methods. This scale demonstrated good reliability (Cronbach α=0.80), with higher scores indicating higher levels of autonomy.

#### Maladaptive Pain Perceptions

Pain-related perceptions were assessed through a 6-weekly questionnaire. Catastrophizing was measured using the validated Pain Catastrophizing Scale (PCS), which consists of 13 items evaluating rumination, magnification, and helplessness [50]. The scale had excellent reliability (Cronbach α=0.90) with a total score ranging from 0 to 52 and higher scores indicating greater degrees of catastrophizing. Fear-avoidance beliefs were assessed using the validated Fear-Avoidance Beliefs Questionnaire [51]. This questionnaire includes two subscales: (1) the FABQA work subscale (FABQW), comprising 7 items (scores ranging from 0 to 42), which measures attitudes about work and its relationship to musculoskeletal concerns, and (2) the FABQA physical activity subscale (FABQA), comprising 4 items (scores ranging from 0 to 24), which evaluates perceptions of movement and physical activities. The FABQW demonstrated good reliability (Cronbach α=0.82), whereas the FABQA demonstrated poor reliability (Cronbach α=0.42). Higher scores on each subscale indicated higher levels of fear-avoidance beliefs.

#### Pain Intensity

Participants indicated their pain intensity on a visual analog scale (VAS), ranging from 0 (“no pain”) to 100 (“worst pain”) daily. The adaption of the VAS to digital platforms, including smartphones, has been validated in previous studies [52].

#### Physical Activity

Daily step count, measured by the Fitbit Inspire, was used to estimate physical activity. Research has demonstrated the accuracy of Fitbit activity trackers in measuring step count in both laboratory and free-living settings [53]. Previous studies have associated step count with physical activity, indicating individuals with <5000 steps per day as sedentary and those with at least 10,000 steps per day as active [54].

#### Personal Experience With the Intervention and Perceived Impact on Health

After the 24-week intervention, semistructured interviews were conducted in 2 parts. The first part explored participants’ perceived impact of the intervention through open-ended questions, while the second part presented illustrations of various app features, prompting participants to discuss their experiences with each one. The interview guide was developed following the qualitative research framework outlined by Braun and Clarke [55]. Two researchers compiled an initial set of questions relevant to the study’s objectives. The questions were structured, progressing from general to more specific topics, and were iteratively refined to ensure clarity and appropriateness. To encourage deeper reflection and uncover underlying thought processes, probing follow-up questions were incorporated, guided by the laddering theory [56]. The final interview guide was piloted with a third researcher.

### Statistical Analysis

#### Sample Size

An a priori power analysis was conducted using G*Power [57] to determine the required sample size. Kristjánsdóttir et al [58] reported a moderate improvement in pain catastrophizing (Cohen *d*=0.74) after a 5-month intervention using a smartphone app for individuals with pain. To detect a moderate effect size at a 5% significance level with a power of 80%, a minimum group of 28 participants was required. Considering a 50% dropout rate, enrolling at least 56 participants was recommended [59].

#### Quantitative Analysis

Quantitative data were analyzed using the SPSS statistical software package (version 28.0; IBM Corp ). Univariate descriptive statistics were conducted to summarize sociodemographic and background variables. Linear multilevel models were used to evaluate changes in pain perceptions (PCS, FABQA, and FABQW), pain intensity (VAS), and physical activity (daily step count) [60]. Given the hierarchical structure of the data, with repeated measurements nested within individuals, this approach accounts for both within-individual and between-individual variation. Random intercepts were included to allow each participant to have a unique baseline value, while random slopes accounted for individual differences in the rate of change over time. In addition, the model estimated the covariance between intercepts and slopes, providing insight into whether baseline values influenced subsequent trajectories. To assess pain intensity and physical activity, the average scores were calculated across 5 periods: 4 periods of 5 consecutive weeks and 1 period of 4 consecutive weeks. Periods with fewer than 5 valid measurements were excluded, and days with fewer than 500 recorded steps were also considered invalid [61,62]. To maximize data inclusion, multilevel models use a full-information maximum likelihood approach, which estimates parameters based on all available data points rather than excluding participants with incomplete data [60].

#### Qualitative Analysis

Qualitative data from the semistructured interviews were analyzed using the Qualitative Analysis Guide of Leuven [63]. This 10-step approach included a preparation phase and a coding phase. First, interviews were transcribed and summarized into brief abstracts. Concrete experiences were transformed into concepts, and these were compared across interviews. Common concepts served as initial codes for the coding process and underwent refinement over several iterations. The final codes were integrated into a meaningful conceptual framework in relation to the research questions. Two researchers conducted the analysis, resolving disagreements through consensus or consultation with a third researcher. To ensure anonymity and maintain neutrality, all participant quotes are presented using the pronoun “he” throughout the paper, regardless of the participant’s sex.

#### Integration of Quantitative and Qualitative Results

Two researchers evaluated the alignment between the quantitative and qualitative results, encompassing confirmation, expansion, or discordance [64]. Disagreements were resolved through consensus or consultation with a third researcher. The metainferences derived from this analysis were presented in a side-by-side joint display for each research question.

## Results

### Participant Characteristics

**Figure 2:**
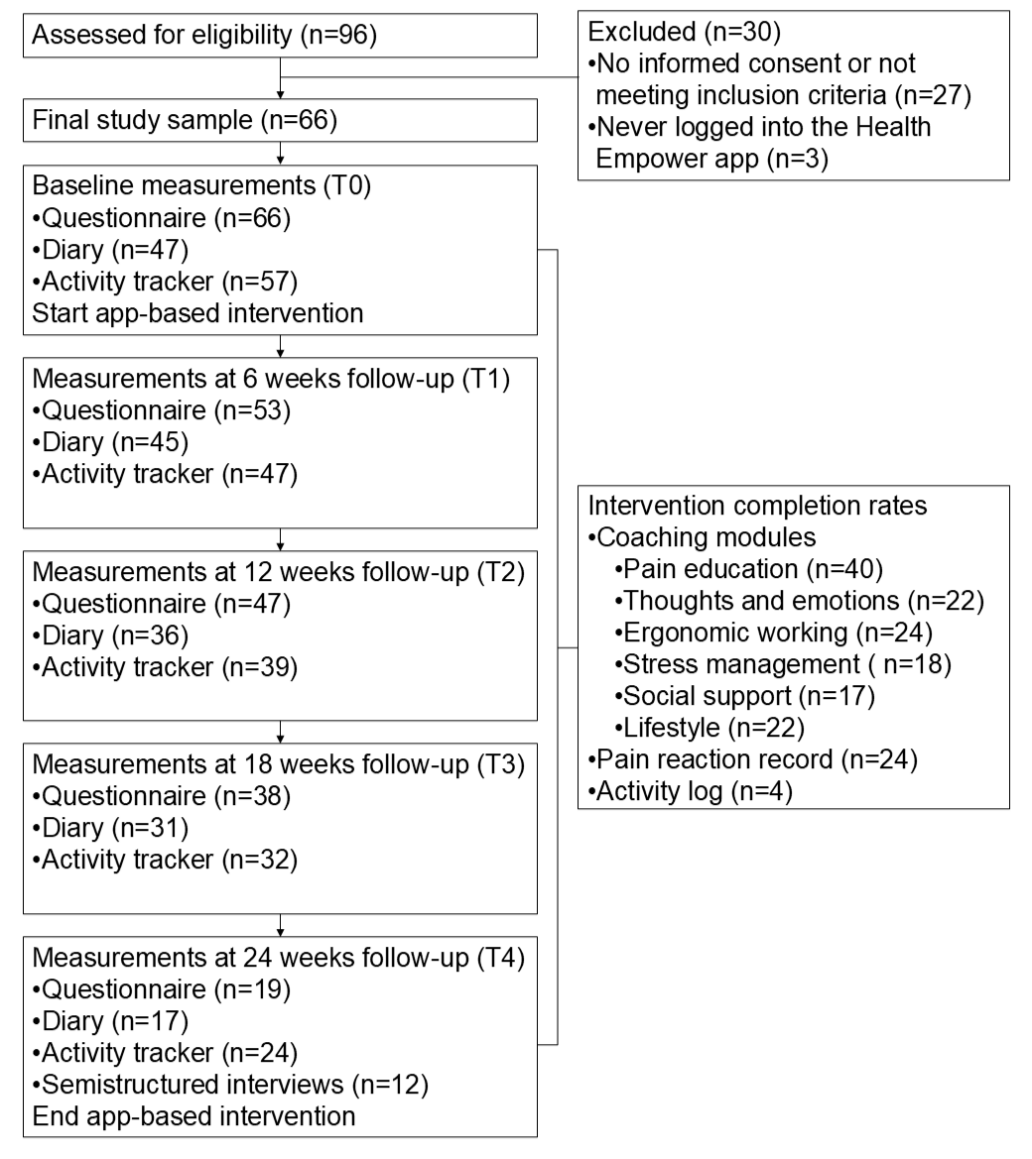
Flowchart of the study.

A total of 96 employees expressed interest in participating in the study (Figure 2). Overall, 27 were excluded for not meeting the inclusion criteria or, failing to provide informed consent and 3 never logging into the app. The remaining 66 employees (n=57, 86% female) were included in this study. An overview of sociodemographic and background information is provided in Table 1. Baseline characteristics were comparable between participants who completed the study (ie, completed the 24-week follow-up questionnaire) and those who dropped out (Multimedia Appendix 2).

**Table 1:** Sociodemographic and baseline information of study participants (n=66).

Characteristics			Values
Age (y), mean (SD)			42 (10.8)
Sex (female), n (%)			57 (86)
Degree (bachelor or higher), n (%)			55 (83)
Profession (health care), n (%)			60 (91)
Tenure (≥10 y), n (%)			37 (56)
Work arrangement (full time), n (%)			35 (53)
Multisite pain (yes), n (%)			59 (89)
Pain duration (≥3 mo), n (%)			61 (92)
Pain-related sick leave (≥1 mo), n (%)			3 (5)
**Work-related factors, mean (SD)**			
	Physical job demand	6.2 (2.7)	
	Workload	9.5 (2.5)	
	Job autonomy	12.9 (3.8)	
	Social support	14.2 (3.5)	
**Maladaptive pain perceptions, mean (SD)**			
	Pain catastrophizing	13.3 (7.7)	
	Fear-avoidance beliefs (activities)	9.3 (4.3)	
	Fear-avoidance beliefs (work)	15.2 (7.4)	
Pain intensity (VAS^a^), mean (SD)			33.0 (18.4)
Physical activity (daily step count), mean (SD)			9564 (2579.3)

^a^VAS: visual analog scale.

### Effect of the App-Based Pain Management Intervention

Metainferences of quantitative and qualitative results on the potential effect of the intervention are presented in Table 2. Overall, there was alignment between the quantitative and qualitative results, with qualitative insights explaining observations in the quantitative data.

**Table 2:** Joint display of quantitative and qualitative findings on the intervention effect.

Theme	Quantitative findings	Qualitative findings	Metainferences
Maladaptive pain perceptions	There was a small but significant decrease in PCS^a^. Variance in regression slopes for FABQA^b^ was significant.	Participants felt reassured by the information. Some adopted a biopsychosocial mindset, while others reinforced a biomedical perspective.	The reduction in PCS aligns with feelings of reassurance. Varying regression slopes for FABQA reflect shifts toward a biopsychosocial or biomedical perspective.
Pain intensity	Variance in regression slopes for VASc was significant.	Participants had varied experiences regarding pain evolution. Pain reduction was often attributed to other interventions.	Varying regression slopes for VAS reflect diverse experiences regarding pain evolution. Qualitative findings expand on the intervention effect by attributing pain reduction to other interventions.
Physical activity	There was no significant change in daily step count.	Most participants reported minimal lifestyle changes. Participants reported increased awareness of activity levels.	No change in step count is consistent with reports of minimal lifestyle changes. Qualitative findings expand on intervention effect by highlighting increased awareness of activity levels.

^a^PCS: Pain Catastrophizing Scale.

^b^FABQA: Fear-Avoidance Beliefs Questionnaire (physical activity subscale).

^c^VAS: visual analog scale.

### Pain Perceptions

Quantitative data analysis showed a decrease in pain catastrophizing (B=−0.83, *P*<.001, *d*=−0.27), reflecting reduced pain-related fear (Table 3; Multimedia Appendix 3). The marginally significant variance in individual slopes for pain catastrophizing (σ²=1.00; *P*=.09) and the negative intercept-slope covariance (σ=−0.38; *P*=.09) suggest that participants with higher baseline catastrophizing tended to show a more pronounced decrease over time. Furthermore, the significant variance in individual slopes for fear-avoidance beliefs regarding physical activities (σ²=1.34; *P*=.04) indicates increasing fear-avoidance beliefs in some participants and decreasing beliefs in others (Table 3; Multimedia Appendix 3).

Qualitative data analysis revealed two subthemes regarding the impact of the intervention on pain perceptions: (1) worries and insecurities and (2) biopsychosocial mindset. Consistent with the observed decrease in pain catastrophizing scores, many participants found reassurance in the information provided by the intervention, feeling that reducing the impact of pain on their lives was possible. This reassurance inspired them to optimize their management strategies. The recognition of their existing coping efforts also provided validation and motivation to continue these strategies. For instance, participant 2 explained how the intervention confirmed that his current pain management approaches were effective, reinforcing his confidence:

When I read the information in the app, I often thought, “Well, yes, I can actually manage my pain this way too.” Sometimes I was already doing it, but it was nice to have confirmation that I wasn’t doing anything wrong and that I should keep going. That information really helped me.

Participant 2

The variance in regression slopes for fear-avoidance beliefs was reflected in the diverse perspectives on the factors contributing to pain and strategies for coping after the intervention. For example, participant 3 continued to emphasize physical factors as the primary contributors to his pain. This participant attributed his pain to past injuries or surgeries and viewed his work environment as an important aggravator:

I had surgery for a cartilage injury, and my joints inflame easily. I don’t believe my pain is psychological. Stress might affect muscle tension, but it’s not as detrimental as wear and tear...My posture isn’t great when I’m working at the computer. I think this has caused muscle imbalances that led to my neck pain.

Participant 3

This focus on physical contributors led participants, such as participant 5, to prioritize strategies aimed at reducing physical strain. For example, this participant adopted avoidance behaviors to prevent further pain:

I’m much more conscious of my movements and posture now. I don’t lift things by bending over anymore. I bend my knees or get down on one knee to pick something up. I learned that from the app.

Participant 5

In contrast, several participants recognized the role of psychosocial factors in their pain, acknowledging that cognitive and emotional processes can influence their pain experience. For instance, participant 7 reflected on how the intervention shifted his perspective from viewing pain primarily as a physical issue to a biopsychosocial phenomenon, helping him recognize the drawbacks of avoidance and the benefits of staying active. Consequently, this participant became less concerned with ergonomics to reduce physical strain:

I didn’t realize how convinced your brain can be that you’ll feel pain during certain activities...One of the things I learned was that staying still is actually bad. But what do many people with pain do? They just sit there, and it only gets worse....I found the idea of stopping complaining about the pain and just getting moving really useful...I didn’t spend much time on the information about lifting, I don’t think it’s that important in my situation.

Participant 7

**Table 3:** Multilevel analysis of pain perceptions, pain intensity, and physical activity.

Parameter		Estimate (SE)	*P* value
**Pain catastrophizing**			
	Fixed effect	−0.83 (0.23)	<.001^a^
	Random intercepts	44.15 (9.09)	<.001
	Random slopes	1.00 (0.59)	.09
	Covariance (intercept-slope)	−0.38 (0.22)	.09
**Fear-avoidance beliefs (activities)**			
	Fixed effect	−0.35 (0.24)	.15
	Random intercepts	8.40 (2.74)	.002
	Random slopes	1.34 (0.64)	.04
	Covariance (intercept-slope)	−0.12 (0.29)	.67
**Fear-avoidance beliefs (work)**			
	Fixed effect	−0.25 (0.26)	.34
	Random intercepts	44.16 (9.70)	<.001
	Random slopes	0.81 (0.71)	.23
	Covariance (intercept-slope)	−0.11 (0.32)	.72
**Pain intensity (VAS^b^)**			
	Fixed effect	−0.37 (0.84)	.67
	Random intercepts	292.60 (66.88)	<.001
	Random slopes	17.29 (8.07)	.03
	Covariance (intercept-slope)	−0.26 (0.19)	.17
**Physical activity (daily step count)**			
	Fixed effect	107.50 (87.48)	.23
	Random intercepts	5,667,049.59 (1,256,486.10)	<.001
	Random slopes	108,345.09 (73,646.87)	.14
	Covariance (intercept-slope)	0.34 (0.33)	.30

^a^Small effect size (Cohen *d*=−0.27).

^b^VAS: visual analog scale.

### Pain Intensity

Quantitative data analysis showed a significant variance in regression slopes for pain intensity scores (σ²=17.29; *P*=.03; Table 3; Multimedia Appendix 3). While pain intensity increased for some participants, it decreased for others.

The qualitative data revealed two subthemes related to pain intensity: (1) evolution of pain and (2) reasons for pain reduction. Consistent with the quantitative data, participants provided diverse perspectives on the evolution of their pain. Many participants expressed disappointment that their pain had not decreased after the intervention. For example, participant 9 was frustrated with the limited impact of the intervention on his pain, noting that it did not provide the actionable tips he had expected:

A lot of people ask me, “Has it helped with the intensity of the pain?” and I have to answer, “No,” because I was expecting more specific tips that I could try out

Participant 9

Despite not experiencing a general improvement in their pain concerns, some participants recognized that the intervention helped reduce the burden of pain in specific situations. For instance, participant 12 shared how the intervention helped him communicate his needs more effectively, thereby reducing stress and pain:

I’ve learned to inform others about my pain problem without complaining. Just a brief “look, I can’t do this alone, I need help because of my neck,” and that reduces my stress and pain...But it’s not gone, of course.

Participant 12

Participants who experienced improvements in their pain often viewed the intervention as secondary to other factors. They believed that changes might have occurred over time or were attributable to other interventions. Participant 6 conveyed this ambiguity, questioning whether the intervention had any real impact on his pain:

I have to say that my pain has decreased...But is it because of the app? I don’t really feel that way. It might just be coincidental since my pain was already starting to decrease, so it could be a combination of both.

Participant 6

### Physical Activity

Quantitative data analysis showed no significant effect of the intervention on daily step count (Table 3; Multimedia Appendix 3).

Two subthemes emerged from the qualitative data regarding the effect of the intervention on physical activity: (1) awareness and (2) physical activity habits. Many participants reported that the intervention heightened their awareness of daily activities, leading them to reflect on their daily routines. For instance, participant 2 described how the daily questionnaires encouraged him to think critically about his activity level:

You had to fill out your questionnaire every evening and reflect on your day...I might have sat down too much or walked a lot...It made me aware that maybe I had done too little or too much. I found that really useful.

Participant 2

This awareness, combined with Fitbit prompts, served as a motivator for some participants to move more and rethink their sedentary habits. Participant 1 explained how tracking his activities encouraged him to be more active:

Both the Fitbit and the app motivate you to think more about your health and to sit less. Since you have to record how long you’ve been sitting and walking, it makes you realize. I never really thought about it before.... Now, I’m more likely to go for a walk or do something else instead of lying on the couch. When you see that you’ve only taken a few steps today, you make sure to walk more the next day.

Participant 1

However, overall changes in physical activity habits were minimal, which is consistent with the quantitative findings. Participants with physically demanding jobs, such as participant 9, reported that the intervention had little impact on their routines:

I have a demanding job and my household responsibilities...I do stand still a lot at work, but I also move around a lot. By the evening, I just don’t feel like doing more. I do check the Fitbit, but it doesn’t motivate me to go outside in the evening to reach ten thousand steps. My life hasn’t changed because of the Fitbit.

Participant 9

### Factors Influencing the Effectiveness of the App-Based Pain Management Intervention

An analysis of the semistructured interviews identified three themes related to factors influencing the effectiveness of the intervention: (1) the intervention quality, (2) the participant’s personal characteristics, and (3) the work context. The metainferences of the quantitative and qualitative results on these factors are presented in Table 4. Overall, there was alignment between the quantitative and qualitative results, with the qualitative insights explaining observations in the quantitative data.

**Table 4:** Joint display of quantitative and qualitative findings on factors influencing effectiveness.

Theme	Quantitative findings	Qualitative findings	Metainferences
Intervention quality	In total, 36% of participants completed each module, 36% used the pain reaction record, and 6% used the activity log. Final follow-up questionnaire was completed by 29% of the participants.	Some found the content clear and useful, while others saw it as too basic or impractical. Interactive elements were well-received, but limited flexibility and integration options hindered engagement.	Nonuse and dropout rates align with mixed perceptions of content relevance and practicality. Nonuse and dropout rates reflect engagement barriers related to intervention design.
Personal characteristics	Little sick leave and favorable baseline scores were reported for pain perceptions, pain intensity, and physical activity. Completion rates were higher for physical-focused modules (35%) compared with psychosocial-focused ones (29%).	Pain concerns were often perceived as manageable, leading to casual engagement with the app. Cognitive biases, including perceived expertise and skepticism, influenced engagement with the app.	Limited impact of pain on life, as reflected by favorable baseline scores, corresponds to high nonuse. Cognitive biases reflect high nonuse and lower completion rates for psychosocial-focused modules compared with physical-focused ones.
Work context	Favorable baseline scores were reported for physical job demands, workload, social support, and job autonomy.	Limited access to adjustable equipment and shared workstations made it difficult to apply ergonomic recommendations. Lack of understanding and support at work made it difficult to implement work modifications and coping strategies.	Despite favorable work-related scores, qualitative findings reveal structural barriers hindering ergonomic adjustments. Despite favorable social support ratings, qualitative findings reveal workplace dynamics hindering coping and modifications.

### Intervention Quality

Follow-up data showed relatively high nonuse and dropout rates (Figure 2). On average, each module was completed by only 24 (36%) participants. The pain reaction record was used by 24 (36%), while the activity planner was used by only 4 (6%) participants. Regarding dropout, 47 participants completed the 12-week follow-up questionnaire 28 (71%), but only 19 (29%) completed the 24-week follow-up.

Two subthemes emerged from the qualitative data regarding the quality of the intervention: (1) content and (2) design. Consistent with the high nonuse and dropout rates, mixed reactions to content and design suggested that the intervention struggled to maintain long-term engagement and meet the diverse needs of its users. Some found the information provided clear, actionable, and accessible. For instance, participant 7 appreciated the straightforward, nonmedical language:

I found the content good and accessible for a broad audience ... not too medical, just very basic but with enough information. And the videos in between were well done.

Participant 7

However, some participants felt the advice was too basic or impractical for their specific situations. Participant 1 highlighted the difficulty of applying certain ergonomic tips because of his use of bifocals:

Tips like “your screen should be at eye level”.... Sorry, that doesn’t work for me because I have bifocals, so my screen needs to be lower to read properly...Also the tips on how to sit properly, I can’t keep that up for two hours.

Participant 1

In addition, some participants, such as participant 6, found that the intervention focused too much on pain, particularly through repetitive questionnaires. This emphasis was counterproductive, making participants more fixated on their pain rather than helping them manage it:

I am not someone who is fixated on pain all the time. Reflecting on pain in the questionnaires was a drawback of this study for me. I didn’t want to always think about pain.

Participant 6

The design of the app, including its usability and visual appeal, also impacted participant engagement. Many participants appreciated the interactive elements, such as videos and quizzes. Familiar design choices, such as chat-style questionnaires, were particularly well-received, as noted by participant 12:

It wasn’t just text, but also videos and quizzes. I enjoyed testing myself to see if I understood everything...The chat-style questionnaire was very intuitive. I buy a lot online so it was recognizable...The colors were neutral, which was easy on the eyes.

Participant 12

Despite these positives, several design elements did not align well with the busy lives of the participants. A lack of flexibility was a common concern. Participant 9 expressed frustration with not being able to advance through the content when he had time available:

It would have been better if there was more flexibility.… It was disappointing that I couldn’t move on when I had some time. Don’t block people, let them explore further. People have limited time because of work. When they find time, let them continue with the modules.

Participant 9

Time-consuming actions and poor integration with other tools also detracted from the experience. Some participants found the app redundant, particularly if they were already using more comprehensive tools. For example, participant 5 preferred his own activity tracker because it featured automated tracking instead of questionnaires:

I have another activity tracker with more options. It automatically tracks heart rate and activities, so I always checked that app.... I mostly used your app to go through the modules, but I didn’t really use the other features because I had something else, and maybe even better.

Participant 5

Furthermore, notifications, while helpful, were often insufficient to maintain regular engagement with the app. Participant 11 suggested that more prominent alerts, similar to those used in social media apps, might have encouraged more consistent use:

What would help me is something like a red dot or a number, like on social media apps...That would remind me that there is something to do. I got the notifications for the questionnaires, but that wasn’t enough to keep me actively engaged.

Participant 11

### Personal Characteristics

Completion rates varied considerably between coaching modules (Figure 2). While 61% (n=40) of participants completed the first module on pain education, the modules focusing on physical topics (ie, modules 3 and 6) had an average completion rate of 35% (n=23), and those on psychosocial topics (ie, modules 2, 4, and 5) had a lower average completion rate of 29% (n=19).

Two subthemes emerged from the qualitative data regarding personal characteristics: (1) the impact of pain on daily life and (2) cognitive biases. The differences in completion rates of coaching modules highlighted how participants’ beliefs and attitudes influenced their engagement with various aspects of the intervention. For many participants, pain had a manageable impact on their daily lives, leading to a more casual engagement with the app. For instance, participant 11, who described his pain as an inconvenience rather than debilitating, participated out of curiosity:

I didn’t have specific expectations about the study. I thought, “Well, if it helps, great; if not, no harm done”.... I have to say, I didn’t complete or use everything. I often thought, “It’s not bad, I’m doing well.” My back pain is unpleasant, but manageable. I live my life; I don’t stay home just because my back hurts.

Participant 11

This mindset aligns with favorable baseline scores on pain perceptions and pain intensity, as well as the relatively high daily step count at baseline and low self-reported sick leave (Table 1).

Furthermore, many participants felt they were already well-versed in pain management, whether through personal experience, previous treatments, or a professional background. This existing knowledge often led to limited engagement with the app, as they did not expect to learn new information. Participant 8 reflected on how much content was a review of what he had already learned:

I found it valuable to check if there were things I didn’t know or could improve...But a lot of it was repetition, as I had already gone through a similar program at a specialized center. So, I skimmed through some parts fairly quickly.

Participant 8

Participants also had different perspectives on the biopsychosocial approach of the intervention. For example, participant 4 appreciated the cognitive approach as it aligned with his ongoing treatment:

I found it interesting because I’m seeing a new physiotherapist who also focuses a lot on how thoughts influence pain and how it’s processed in the brain...So, this aligned with that approach, especially since no clear cause has been found for my pain. I have a muscle condition, but I’m not convinced that it explains all my symptoms.

Participant 4

In contrast, others were skeptical of the cognitive and psychosocial elements, linking their pain to biomedical factors. Consequently, these participants favored physical approaches to pain management. For example, participant 3 expressed doubt about the effectiveness of cognitive strategies for his pain, limiting his engagement with those aspects of the content:

With my background and education, I’m skeptical about the cognitive approach to pain...I didn’t have high expectations for improvement through this method, as I usually know what’s causing my pain, muscle imbalance or a damaged joint. I’m more inclined to address pain by exercising.... I still read everything in the coaching, but I paid more attention to some things than others.

Participant 3

### Work Context

Overall, participants reported favorable scores for physical job demands, workload, social support, and job autonomy at baseline, indicating a good balance between job demands and job resources (Table 1).

Two subthemes emerged from the qualitative data regarding work context: (1) ergonomic equipment and (2) social support. Despite the favorable scores on work-related factors, several participants encountered barriers when trying to implement advice from the app. Some participants, such as participant 5, were unable to customize their workspaces because of shared workstations and a lack of adjustable equipment, which often resulted in discomfort or exacerbated pain:

We have five different workstations, and at stations three and four, the tables are lower, which is harder for me because I have to bend over and lift patients from a lower position. I notice that on those stations, I have more complaints.... I can’t do much about it because those tables have standard heights.

Participant 5

Many participants reported that persistent pain often received less recognition compared with more visible health conditions. This lack of understanding and empathy made it difficult for them to openly discuss their pain and request necessary accommodations. Participant 11 expressed frustration with the dismissive attitudes of some colleagues:

Most people don’t take it seriously when you say you’re in pain. They might listen, but they quickly forget. It’s the usual “Just take a pill and keep going.”…. There’s just a lack of understanding, and I’ve tried to accept it because they don’t get it. If you don’t have pain yourself, it’s hard to understand.

Participant 11

In addition, resistance from colleagues and employers toward modifying work conditions was a recurring issue. This resistance often stemmed from concerns about creating distinctions between employees or fears of reduced productivity. Participant 8 noted the difficulties he faced when returning to work after a long absence because of pain:

They don’t adjust anything; it’s really hard to deal with...I was off work for a year, and when I returned, the first thing they said was that I had to do all the work - all kinds of patients, all shifts...I think just an extra ten-minute break would help, but it’s really hard to get that. They don’t like it because then the other colleagues have to work harder or cover for me.

Participant 8

However, efforts made by colleagues and supervisors to optimize the work environment and reduce physical demands were much appreciated by the participants. These efforts often included providing ergonomic equipment or adjusting work schedules and tasks. For instance, participant 2 highlighted the accommodations made to help manage his chronic pain:

I feel like they make an effort to keep people with chronic pain employed...They created a schedule for me where I work three half-days, one full day, and have one day off. They’ve also tried to ensure I have a variety of tasks, so I can’t say they aren’t taking it into account.

Participant 2

## Discussion

### Effect of the App-Based Pain Management Intervention

This study evaluated an app-based intervention designed to support employees with musculoskeletal pain by addressing maladaptive pain perceptions, providing work-related guidance, and promoting healthy activity habits.

Consistent with previous research demonstrating that app-based pain management can effectively reduce pain catastrophizing in patient populations, this study suggests that such an approach can also benefit employees [16-18]. The significant reduction in pain catastrophizing scores aligned with participants’ reports of feeling reassured. By focusing on pain neuroscience education to shift perceptions of pain from a structural or injury-based model toward a broader understanding of pain mechanisms, participants may have gained a greater sense of control, thereby reducing the perceived threat of pain [31,65]. However, the intervention did not result in significant overall improvements in fear-avoidance beliefs, diverging from previous findings [16]. The significant variability in regression slopes for fear-avoidance beliefs suggests that, for some participants, the intervention reinforced their focus on physical contributors to pain.

Similarly, while prior studies have shown that mHealth interventions can effectively reduce pain intensity, our intervention did not yield a significant overall effect [15]. Moreover, the significant variability in pain trajectories suggests that some participants experienced increased discomfort.

Despite incorporating strategies for graded activity, personal goal setting, and healthy lifestyle habits, the intervention did not lead to significant increases in daily step count [36,41,45,46]. While many participants reported greater awareness of their movement habits, this did not translate into behavioral change. This contrasts with prior studies reporting small to moderate increases in physical activity following mHealth interventions, even without direct human support [66].

### Factors Influencing the Effectiveness of the App-Based Pain Management Intervention

The content of the intervention likely influenced its effectiveness. Unlike app-based pain management interventions that integrate structured exercise programs, our intervention primarily focused on education [16]. While modifying cognitive representations of pain can positively impact pain experiences, research suggests that education should be combined with exercise therapy to achieve meaningful reductions in pain and disability [65,67,68]. Moreover, exercise programs also provide positive movement experiences, which can help reframe fearful cognitive representations of pain [36,67]. Combining education with progressive exercise may explain why Sitges et al [16] observed significant improvements in fear-avoidance beliefs following app-based pain management. In addition, our intervention provided general ergonomic recommendations, which may not have adequately addressed the specific challenges faced by some employees in physically demanding jobs [69,70]. Prior research has highlighted that physically demanding jobs exhibit greater variability in workstation design compared with office-based roles, making broad ergonomic guidance less effective [71].

Beyond content, design-related factors might have influenced the intervention’s effectiveness. Some participants reported that self-monitoring heightened vigilance toward pain. Research has shown that heightened attention to pain can reinforce avoidance behavior and amplify pain perception, potentially explaining the variability in regression slopes for fear-avoidance beliefs and pain intensity [20,32]. In addition, the absence of gamification elements, such as leaderboards and rewards, could explain the limited effect of the intervention on physical activity [72]. A systematic review found that social features, in particular, are effective in promoting physical activity [73]. However, individuals with chronic musculoskeletal conditions often prefer privacy in self-management apps and may be reluctant to engage with social features [74]. Furthermore, several design choices hindered engagement with our intervention, potentially hindering its effectiveness. Participants expressed frustration with restrictive features such as time locks, which prevented them from progressing through content at their own pace. There was also a clear demand for more seamless and automated functionality, particularly for activity tracking. Given the rapid evolution of digital technologies, users have increasingly high expectations for convenience and personalization [75]; when interventions are perceived as cumbersome or misaligned with individual preferences, the likelihood of disengagement increases.

Individual differences also shaped engagement with the intervention. Many participants reported that pain had a limited impact on their daily lives, which may have contributed to high nonuse rates. In addition, some employees selectively attended to information that aligned with their preexisting beliefs while disregarding content that challenged them, limiting the potential for meaningful change [76,77]. This confirmation bias may be particularly relevant for those with strong fear-avoidance beliefs, who may have used the intervention to validate rather than modify their perceptions of pain. Cognitive biases frequently contribute to errors in clinical decision-making among health care professionals [78]. Moreover, research has shown that biomedical perspectives remain dominant in health care despite the growing emphasis on psychosocial factors in medical education [79-81]. Raising awareness of cognitive biases, combined with simple communication and counterexplanation, can foster critical thinking and openness to new perspectives [82,83]. While pain neuroscience education already integrates some of these strategies, individuals in the precontemplation stage of behavior change (eg, participants who consider themselves experts on their condition) may require additional guidance [31,84].

Finally, the broader workplace context also played a role in the intervention’s effectiveness. Participants highlighted several workplace challenges, including a lack of ergonomic equipment and resistance from colleagues and supervisors to modifying work conditions. These challenges may have contributed to the significant variability in regression slopes of fear-avoidance beliefs and pain intensity, as participants who perceived a conflict between the intervention’s recommendations and their work environment might have experienced worsening scores. Prior research suggests that nonsupportive work environments can undermine pain management interventions, particularly when colleagues or supervisors question the legitimacy of work modifications or coping strategies [19,69,85,86].

### Study Contributions and Future Directions

This was the first study to evaluate an app-based pain management intervention specifically designed for employees. Our findings indicate that mHealth can support the multidimensional treatment of musculoskeletal pain in the workplace by reducing the perceived threat of pain, an important barrier to work participation [19,20,22-24]. Moreover, addressing maladaptive pain perceptions and increasing awareness of activity habits are essential steps in initiating active coping strategies, such as physical exercise, which can improve physical functioning [34,36,87]. These findings suggest that app-based pain management interventions may offer accessible and scalable support for some employees, reducing reliance on intensive face-to-face care. This, in turn, could enable health care providers to focus time and resources on employees at higher risk of long-term disability.

Our results also highlight the value of self-monitoring in capturing the evolution of risk factors over time. In line with prior research, our results suggest that maladaptive pain perceptions may emerge over time as employees continue to struggle with managing pain in the workplace [88]. Therefore, conducting assessments only at the onset of pain concerns may overlook employees whose risk of disability develops gradually over time. Continuous self-monitoring with mobile apps could facilitate the early identification of these at-risk employees. However, self-monitoring tools should be carefully designed to avoid reinforcing hypervigilance to pain signals. Future studies should explore optimal monitoring frequency or investigate automated tracking of physiological parameters as a less intrusive alternative. For instance, a systematic review has demonstrated significant associations between self-reported pain intensity and physiological markers, such as heart rate variability and skin conductance [89].

Despite the potential benefits of mHealth, our findings suggest that digital interventions alone are unlikely to meet the needs of all employees with musculoskeletal pain. Many participants required more tailored advice and additional support to initiate and sustain new coping strategies. This highlights the need for blended interventions that combine digital tools with professional guidance [90]. Research shows that face-to-face ergonomic consultations are more effective than generic self-help materials in reducing musculoskeletal symptoms [91]. Likewise, health care providers can enhance the effectiveness of pain neuroscience education by tailoring it to the individual’s context and using empathic communication [65,67,92]. Previous studies have successfully combined digital learning modules with in-person consultations, enabling participants to review educational materials at their own pace while using face-to-face sessions to clarify misconceptions and personalize treatment strategies [93,94].

Finally, our results underscore the importance of embedding pain management interventions within workplace policies. Several participants encountered barriers that limited the implementation of the app’s recommendations, including a lack of ergonomic equipment and resistance from colleagues or supervisors. Research suggests that successful return-to-work interventions foster a supportive workplace culture by educating teams about the challenges of chronic pain and involving them in defining appropriate work modifications [27,69,85,95,96]. Occupational services for prevention and protection at work could play a key role by using mHealth data to provide targeted guidance to managers, increasing their willingness and ability to support health-related interventions [70].

### Study Limitations

Several limitations of this study should be acknowledged. First, the 1-group pilot design limits causal inference regarding the effectiveness of the app-based pain management intervention. Nevertheless, the integration of quantitative and qualitative data provided a rich understanding of how participants engaged with the intervention and perceived its impact [97]. These insights are valuable for refining the intervention and informing the design of a future large-scale clinical trial [30].

Second, the study sample consisted of employees with persistent musculoskeletal pain who remained in active employment. These participants reported relatively favorable baseline scores for pain intensity, pain perceptions, and physical activity, which may have reduced the observed effectiveness of the app-based intervention. However, the significant variance in individual slopes for pain intensity suggests that the intervention may have been beneficial for a subset of participants. Furthermore, our results also revealed a negative intercept-slope covariance for pain catastrophizing, indicating that participants with higher baseline levels of catastrophizing experienced more pronounced improvements over time. This is consistent with findings from the study by Sitges et al [16], which observed greater improvements among participants with higher baseline fear-avoidance beliefs, and with the study by Mönninghoff et al [66], which reported stronger effects of mHealth interventions in individuals with lower initial physical activity levels. Recruitment through the employer may partly explain the favorable baseline characteristics. Employees with more severe musculoskeletal pain may have been less inclined to participate because of privacy concerns despite IDEWE’s strict data protection policies. In addition, recruitment via work-related channels may have inadvertently excluded employees on sick leave, who may report more pronounced maladaptive pain perceptions and less favorable work conditions.

Third, the overrepresentation of health care workers in our sample limits the generalizability of our findings to other sectors [98]. Future studies should explore the effectiveness of mHealth for musculoskeletal pain in more diverse workplace populations, particularly among employees with lower digital health literacy, who may require different communication strategies and additional support [92,99,100].

High dropout and nonuse attrition rates were also a limitation of this study. Although multilevel analysis was used to address missing data, attrition may have influenced the findings [60]. Importantly, discontinuation of app use does not necessarily indicate intervention failure. Some participants may have stopped engaging after they had adopted new coping strategies or reached their personal goals [75]. Future studies should aim to determine the minimum exposure needed to achieve meaningful benefits and establish clearer criteria for evaluating dropout and nonuse attrition.

Finally, the measurement of physical activity warrants consideration. While daily step count is a widely used metric, it does not capture activity intensity [54]. In addition, wrist-worn trackers, such as the Fitbit Inspire, may not accurately record activities that do not involve vertical arm movements, such as cycling [101]. This may have obscured changes in physical activity related to the intervention. Future studies should consider alternative methods for quantifying physical activity levels, such as measuring minutes of moderate and vigorous activity or total energy expenditure [66,101].

### Conclusions

This pilot study demonstrates the potential of an app-based intervention to support employees with musculoskeletal pain by reducing maladaptive pain perceptions and promoting active coping strategies. While the intervention showed promising effects, particularly in reducing pain catastrophizing, its overall impact on fear-avoidance beliefs, pain intensity, and physical activity was limited. The findings underscore the value of mHealth as a low-threshold solution for some employees while also highlighting the need for blended approaches that incorporate professional guidance for those requiring additional support. Moreover, the results emphasize the importance of embedding pain management strategies within workplace policies, as nonsupportive work environments might hinder treatment efficacy. Together, these insights offer valuable guidance for the development of more personalized, scalable, and context-sensitive interventions aimed at improving self-management and work participation among employees with musculoskeletal pain.

## Appendix

Multimedia Appendix 1 Exploratory factor analysis of the Dutch Musculoskeletal Questionnaire.

Multimedia Appendix 2 Baseline comparison between study completers and dropouts.

Multimedia Appendix 3 Regression slopes of outcome variables.

## Abbreviations

FABQA: Fear-Avoidance Beliefs Questionnaire (physical activity subscale)
FABQW: Fear-Avoidance Beliefs Questionnaire (work subscale)
mHealth: mobile health
PCS: Pain Catastrophizing Scale
VAS: visual analog scale

## References

[1] de Kok J, Vroonhof P, Snijders J, Roullis G, Clarke M, Peereboom K, van Dorst P, Isusi I (2019). Work-related musculoskeletal disorders: prevalence, costs and demographics in the EU. European Agency for Safety and Health at Work.

[2] Macchia L, Delaney L, Daly M (2024). Global pain levels before and during the COVID-19 pandemic. Econ Hum Biol.

[3] Cammarota A (2007). The commissions initiative on WRMSDs second phase consultation of European social partners and future developments. Communication and Information Resource Centre for Administrations, Businesses and Citizens.

[4] Statistieken van de uitkeringen over 2022. Rijksinstituut voor ziekte- en invaliditeitsverzekering.

[5] Gonzales CM Council Directive 89/391/EEC of 12 June 1989 on the introduction of measures to encourage improvements in the safety and health of workers at work. The Council of the European Communities.

[6] Colosio C, Mandic-Rajcevic S, Godderis L, van der Laan G, Hulshof C, van Dijk F (2017). Workers' health surveillance: implementation of the Directive 89/391/EEC in Europe. Occup Med (Lond).

[7] Lambrechts MC, Ketterer F, Symons L, Mairiaux P, Peremans L, Remmen R, Vanmeerbeek M, Godderis L (2015). The approach taken to substance abuse by occupational physicians: a qualitative study on influencing factors. J Occup Environ Med.

[8] Walters D, Johnstone R, Bluff E, Jørgen Limborg H, Gensby U (2022). Prevention services for occupational safety and health in the European Union: anachronisms or supports for better practice?. Saf Sci.

[9] Choinière M, Peng P, Gilron I, Buckley N, Williamson O, Janelle-Montcalm A, Baerg K, Boulanger A, Di Renna T, Finley GA, Intrater H, Lau B, Pereira J (2020). Accessing care in multidisciplinary pain treatment facilities continues to be a challenge in Canada. Reg Anesth Pain Med.

[10] Benahmed N, Jonckheer P, Zeevaert R, Vos B, Kohn L (2024). Assessing and monitoring waiting times in healthcare: how to proceed in Belgium?. Belgian Health Care Knowledge Centre (KCE).

[11] Lynch ME, Campbell F, Clark AJ, Dunbar MJ, Goldstein D, Peng P, Stinson J, Tupper H (2008). A systematic review of the effect of waiting for treatment for chronic pain. Pain.

[12] Steinhubl SR, Muse ED, Topol EJ (2015). The emerging field of mobile health. Sci Transl Med.

[13] Rowland SP, Fitzgerald JE, Holme T, Powell J, McGregor A (2020). What is the clinical value of mHealth for patients?. NPJ Digit Med.

[14] Lalloo C, Jibb LA, Rivera J, Agarwal A, Stinson JN (2015). "There's a pain app for that": review of patient-targeted smartphone applications for pain management. Clin J Pain.

[15] Moreno-Ligero M, Moral-Munoz JA, Salazar A, Failde I (2023). mHealth intervention for improving pain, quality of life, and functional disability in patients with chronic pain: systematic review. JMIR Mhealth Uhealth.

[16] Sitges C, Terrasa JL, García-Dopico N, Segur-Ferrer J, Velasco-Roldán O, Crespí-Palmer J, González-Roldán AM, Montoya P (2022). An educational and exercise mobile phone-based intervention to elicit electrophysiological changes and to improve psychological functioning in adults with nonspecific chronic low back pain (BackFit App): nonrandomized clinical trial. JMIR Mhealth Uhealth.

[17] Morcillo-Muñoz Y, Sánchez-Guarnido AJ, Calzón-Fernández S, Baena-Parejo I (2022). Multimodal chronic pain therapy for adults via smartphone: randomized controlled clinical trial. J Med Internet Res.

[18] Mascaro JS, Singh V, Wehrmeyer K, Scott B, Juan J, McKenzie-Brown AM, Lane OP, Haack C (2021). Randomized, wait-list-controlled pilot study of app-delivered mindfulness for patients reporting chronic pain. Pain Rep.

[19] Grant M, O-Beirne-Elliman J, Froud R, Underwood M, Seers K (2019). The work of return to work. Challenges of returning to work when you have chronic pain: a meta-ethnography. BMJ Open.

[20] Vlaeyen JW, Linton SJ (2000). Fear-avoidance and its consequences in chronic musculoskeletal pain: a state of the art. Pain.

[21] Picavet HS, Vlaeyen JW, Schouten JS (2002). Pain catastrophizing and kinesiophobia: predictors of chronic low back pain. Am J Epidemiol.

[22] Carlesso LC, Raja Rampersaud Y, Davis AM (2018). Clinical classes of injured workers with chronic low back pain: a latent class analysis with relationship to working status. Eur Spine J.

[23] Macías-Toronjo I, Rojas-Ocaña MJ, Sánchez-Ramos JL, García-Navarro EB (2020). Pain catastrophizing, kinesiophobia and fear-avoidance in non-specific work-related low-back pain as predictors of sickness absence. PLoS One.

[24] Steenstra IA, Munhall C, Irvin E, Oranye N, Passmore S, Van Eerd D, Mahood Q, Hogg-Johnson S (2017). Systematic review of prognostic factors for return to work in workers with sub acute and chronic low back pain. J Occup Rehabil.

[25] Keyaerts S, Godderis L, Delvaux E, Daenen L (2022). The association between work-related physical and psychosocial factors and musculoskeletal disorders in healthcare workers: moderating role of fear of movement. J Occup Health.

[26] Keyaerts S, Godderis L, Vanden Abeele V, Daenen L (2024). Identifying pain profiles in employees including work-related factors and pain perceptions: a cross-sectional study in Belgian companies. BMJ Open.

[27] Cullen KL, Irvin E, Collie A, Clay F, Gensby U, Jennings PA, Hogg-Johnson S, Kristman V, Laberge M, McKenzie D, Newnam S, Palagyi A, Ruseckaite R, Sheppard DM, Shourie S, Steenstra I, Van Eerd D, Amick BC (2018). Effectiveness of workplace interventions in return-to-work for musculoskeletal, pain-related and mental health conditions: an update of the evidence and messages for practitioners. J Occup Rehabil.

[28] Duffy A, Christie GJ, Moreno S (2022). The challenges toward real-world implementation of digital health design approaches: narrative review. JMIR Hum Factors.

[29] Voorheis P, Zhao A, Kuluski K, Pham Q, Scott T, Sztur P, Khanna N, Ibrahim M, Petch J (2022). Integrating behavioral science and design thinking to develop mobile health interventions: systematic scoping review. JMIR Mhealth Uhealth.

[30] Kistin C, Silverstein M (2015). Pilot studies: a critical but potentially misused component of interventional research. JAMA.

[31] Nijs J, Paul van Wilgen C, Van Oosterwijck J, van Ittersum M, Meeus M (2011). How to explain central sensitization to patients with 'unexplained' chronic musculoskeletal pain: practice guidelines. Man Ther.

[32] Garland EL (2012). Pain processing in the human nervous system: a selective review of nociceptive and biobehavioral pathways. Prim Care.

[33] Vitoula KV, Venneri A, Varrassi G, Paladini A, Sykioti P, Adewusi J, Zis P (2018). Behavioral therapy approaches for the management of low back pain: an up-to-date systematic review. Pain Ther.

[34] Meeus M, Nijs J, Van Wilgen P, Noten S, Goubert D, Huijnen I (2016). Moving on to movement in patients with chronic joint pain. Pain.

[35] Stock SR, Nicolakakis N, Vézina N, Vézina M, Gilbert L, Turcot A, Sultan-Taïeb H, Sinden K, Denis M, Delga C, Beaucage C (2018). Are work organization interventions effective in preventing or reducing work-related musculoskeletal disorders? A systematic review of the literature. Scand J Work Environ Health.

[36] Blickenstaff C, Pearson N (2016). Reconciling movement and exercise with pain neuroscience education: a case for consistent education. Physiother Theory Pract.

[37] Ryan CG, Gray HG, Newton M, Granat MH (2010). Pain biology education and exercise classes compared to pain biology education alone for individuals with chronic low back pain: a pilot randomised controlled trial. Man Ther.

[38] Robertson IT, Cooper CL, Sarkar M, Curran T (2015). Resilience training in the workplace from 2003 to 2014: a systematic review. J Occup Organ Psychol.

[39] Joyce S, Shand F, Tighe J, Laurent SJ, Bryant RA, Harvey SB (2018). Road to resilience: a systematic review and meta-analysis of resilience training programmes and interventions. BMJ Open.

[40] Nijs J, D'Hondt E, Clarys P, Deliens T, Polli A, Malfliet A, Coppieters I, Willaert W, Tumkaya Yilmaz S, Elma ?, Ickmans K (2020). Lifestyle and chronic pain across the lifespan: an inconvenient truth?. PM R.

[41] Bull FC, Al-Ansari SS, Biddle S, Borodulin K, Buman MP, Cardon G, Carty C, Chaput J, Chastin S, Chou R, Dempsey PC, DiPietro L, Ekelund U, Firth J, Friedenreich CM, Garcia L, Gichu M, Jago R, Katzmarzyk PT, Lambert E, Leitzmann M, Milton K, Ortega FB, Ranasinghe C, Stamatakis E, Tiedemann A, Troiano RP, van der Ploeg HP, Wari V, Willumsen JF (2020). World Health Organization 2020 guidelines on physical activity and sedentary behaviour. Br J Sports Med.

[42] Sullivan MJ, Adams H, Rhodenizer T, Stanish WD (2006). A psychosocial risk factor--targeted intervention for the prevention of chronic pain and disability following whiplash injury. Phys Ther.

[43] Szymanski M, Abeele VV, Verbert K (2022). Explaining health recommendations to lay users: The dos and dont's. Proceedings of the ACM IUI Workshops 2022.

[44] Szymanski M, Conati C, Abeele VV, Verbert K (2023). Designing and personalising hybrid multi-modal health explanations for lay users. Proceedings of the 10th Joint Workshop on Interfaces and Human Decision Making for Recommender.

[45] Lindström I, Ohlund C, Eek C, Wallin L, Peterson LE, Fordyce WE, Nachemson AL (1992). The effect of graded activity on patients with subacute low back pain: a randomized prospective clinical study with an operant-conditioning behavioral approach. Phys Ther.

[46] Swann C, Jackman PC, Lawrence A, Hawkins RM, Goddard SG, Williamson O, Schweickle MJ, Vella SA, Rosenbaum S, Ekkekakis P (2023). The (over)use of SMART goals for physical activity promotion: a narrative review and critique. Health Psychol Rev.

[47] Hildebrandt VH, Bongers PM, van Dijk FJ, Kemper HC, Dul J (2001). Dutch Musculoskeletal Questionnaire: description and basic qualities. Ergonomics.

[48] Notelaers G, de Witte H, van Veldhoven M, Vermunt J (2007). Construction and validation of the short inventory to monitor psychosocial hazards. Médecine Travail Ergonom.

[49] van Veldhoven MJ, Prins J, van der Laken PA, Dijkstra L (2015). QEEW2.0: 42 Short Scales for Survey Research on Work, Well-Being and Performance.

[50] Sullivan MJ, Bishop SR, Pivik J (1995). The pain catastrophizing scale: development and validation. Psychol Assess.

[51] Waddell G, Newton M, Henderson I, Somerville D, Main C (1993). A Fear-Avoidance Beliefs Questionnaire (FABQ) and the role of fear-avoidance beliefs in chronic low back pain and disability. Pain.

[52] Delgado DA, Lambert BS, Boutris N, McCulloch PC, Robbins AB, Moreno MR, Harris JD (2018). Validation of digital visual analog scale pain scoring with a traditional paper-based visual analog scale in adults. JAAOS Glob Res Rev.

[53] Fuller D, Colwell E, Low J, Orychock K, Tobin MA, Simango B, Buote R, Van Heerden D, Luan H, Cullen K, Slade L, Taylor NG (2020). Reliability and validity of commercially available wearable devices for measuring steps, energy expenditure, and heart rate: systematic review. JMIR Mhealth Uhealth.

[54] Tudor-Locke C (2010). Steps to better cardiovascular health: how many steps does it take to achieve good health and how confident are we in this number?. Curr Cardiovasc Risk Rep.

[55] Braun V, Clarke V (2016). Successful Qualitative Research: A Practical Guide for Beginners.

[56] Reynolds TJ, Gutman J (1988). Laddering theory, method, analysis, and interpretation. J Advert Res.

[57] Faul F, Erdfelder E, Lang A, Buchner A (2007). G*Power 3: a flexible statistical power analysis program for the social, behavioral, and biomedical sciences. Behav Res Methods.

[58] Kristjánsdóttir ÓB, Fors EA, Eide E, Finset A, Stensrud TL, van Dulmen S, Wigers SH, Eide H (2013). A smartphone-based intervention with diaries and therapist-feedback to reduce catastrophizing and increase functioning in women with chronic widespread pain: randomized controlled trial. J Med Internet Res.

[59] Meyerowitz-Katz G, Ravi S, Arnolda L, Feng X, Maberly G, Astell-Burt T (2020). Rates of attrition and dropout in app-based interventions for chronic disease: systematic review and meta-analysis. J Med Internet Res.

[60] Field A (2018). Discovering Statistics Using IBM SPSS Statistics. 5th edition.

[61] Yao J, Tan CS, Lim N, Tan J, Chen C, Müller-Riemenschneider F (2021). Number of daily measurements needed to estimate habitual step count levels using wrist-worn trackers and smartphones in 212,048 adults. Sci Rep.

[62] Tang LM, Meyer J, Epstein DA, Bragg K, Engelen L, Bauman A, Kay J (2018). Defining adherence: making sense of physical activity tracker data. Proc ACM Interact Mob Wearable Ubiquitous Technol.

[63] Dierckx de Casterlé B, Gastmans C, Bryon E, Denier Y (2012). QUAGOL: a guide for qualitative data analysis. Int J Nurs Stud.

[64] Guetterman TC, Fetters MD, Creswell JW (2015). Integrating quantitative and qualitative results in health science mixed methods research through joint displays. Ann Fam Med.

[65] Watson JA, Ryan CG, Cooper L, Ellington D, Whittle R, Lavender M, Dixon J, Atkinson G, Cooper K, Martin DJ (2019). Pain neuroscience education for adults with chronic musculoskeletal pain: a mixed-methods systematic review and meta-analysis. J Pain.

[66] Mönninghoff A, Kramer JN, Hess AJ, Ismailova K, Teepe GW, Tudor Car L, Müller-Riemenschneider F, Kowatsch T (2021). Long-term effectiveness of mHealth physical activity interventions: systematic review and meta-analysis of randomized controlled trials. J Med Internet Res.

[67] Malfliet A, Ickmans K, Huysmans E, Coppieters I, Willaert W, Bogaert WV, Rheel E, Bilterys T, Wilgen PV, Nijs J (2019). Best evidence rehabilitation for chronic pain part 3: low back pain. J Clin Med.

[68] Siddall B, Ram A, Jones MD, Booth J, Perriman D, Summers SJ (2022). Short-term impact of combining pain neuroscience education with exercise for chronic musculoskeletal pain: a systematic review and meta-analysis. Pain.

[69] Driessen MT, Groenewoud K, Proper KI, Anema JR, Bongers PM, van der Beek AJ (2010). What are possible barriers and facilitators to implementation of a Participatory Ergonomics programme?. Implement Sci.

[70] Rothmore P, Aylward P, Karnon J (2015). The implementation of ergonomics advice and the stage of change approach. Appl Ergon.

[71] Sundstrup E, Seeberg KG, Bengtsen E, Andersen LL (2020). A systematic review of workplace interventions to rehabilitate musculoskeletal disorders among employees with physical demanding work. J Occup Rehabil.

[72] Xu L, Shi H, Shen M, Ni Y, Zhang X, Pang Y, Yu T, Lian X, Yu T, Yang X, Li F (2022). The effects of mHealth-based gamification interventions on participation in physical activity: systematic review. JMIR Mhealth Uhealth.

[73] Tu R, Hsieh P, Feng W (2021). Walking for fun or for “likes”? The impacts of different gamification orientations of fitness apps on consumers’ physical activities. Sport Manag Rev.

[74] Geuens J, Geurts L, Swinnen TW, Westhovens R, Vanden Abeele V (2019). Mobile health features supporting self-management behavior in patients with chronic arthritis: mixed-methods approach on patient preferences. JMIR Mhealth Uhealth.

[75] Eysenbach G (2005). The law of attrition. J Med Internet Res.

[76] Balakrishnan K, Arjmand EM (2019). The impact of cognitive and implicit bias on patient safety and quality. Otolaryngol Clin North Am.

[77] Anderson CA, Lepper MR, Ross L (1980). Perseverance of social theories: the role of explanation in the persistence of discredited information. J Pers Soc Psychol.

[78] Kunitomo K, Harada T, Watari T (2022). Cognitive biases encountered by physicians in the emergency room. BMC Emerg Med.

[79] Jaini PA, Lee JS (2015). A review of 21st century utility of a biopsychosocial model in United States medical school education. J Lifestyle Med.

[80] Setchell J, Costa N, Ferreira M, Makovey J, Nielsen M, Hodges PW (2017). Individuals' explanations for their persistent or recurrent low back pain: a cross-sectional survey. BMC Musculoskelet Disord.

[81] van Dijk H, Köke AJ, Elbers S, Mollema J, Smeets RJ, Wittink H (2023). Physiotherapists using the biopsychosocial model for chronic pain: barriers and facilitators-a scoping review. Int J Environ Res Public Health.

[82] Siebert J, Siebert JU (2023). Effective mitigation of the belief perseverance bias after the retraction of misinformation: awareness training and counter-speech. PLoS One.

[83] Kountz DS (2009). Strategies for improving low health literacy. Postgrad Med.

[84] Prochaska JO, Velicer WF (1997). The transtheoretical model of health behavior change. Am J Health Promot.

[85] Yarker J, Lewis R, Sinclair A, Michlig G, Munir F (2022). Meta-synthesis of qualitative research on the barriers and facilitators to implementing workplace mental health interventions. SSM Ment Health.

[86] Coole C, Watson PJ, Drummond A (2010). Low back pain patients' experiences of work modifications; a qualitative study. BMC Musculoskelet Disord.

[87] Nijs J, Lluch Girbés E, Lundberg M, Malfliet A, Sterling M (2015). Exercise therapy for chronic musculoskeletal pain: innovation by altering pain memories. Man Ther.

[88] Bunzli S, Smith A, Schütze R, Lin I, O'Sullivan P (2017). Making sense of low back pain and pain-related fear. J Orthop Sports Phys Ther.

[89] Avila FR, McLeod CJ, Huayllani MT, Boczar D, Giardi D, Bruce CJ, Carter RE, Forte AJ (2021). Wearable electronic devices for chronic pain intensity assessment: a systematic review. Pain Pract.

[90] Van Daele T, Gerard S, Belmont A (2024). Digital interventions and apps for mental health. Superior Health Council.

[91] Pillastrini P, Mugnai R, Farneti C, Bertozzi L, Bonfiglioli R, Curti S, Mattioli S, Violante FS (2007). Evaluation of two preventive interventions for reducing musculoskeletal complaints in operators of video display terminals. Phys Ther.

[92] Nijs J, Wijma AJ, Willaert W, Huysmans E, Mintken P, Smeets R, Goossens M, van Wilgen CP, Van Bogaert W, Louw A, Cleland J, Donaldson M (2020). Integrating motivational interviewing in pain neuroscience education for people with chronic pain: a practical guide for clinicians. Phys Ther.

[93] Malfliet A, Kregel J, Meeus M, Roussel N, Danneels L, Cagnie B, Dolphens M, Nijs J (2018). Blended-learning pain neuroscience education for people with chronic spinal pain: randomized controlled multicenter trial. Phys Ther.

[94] Toonders SA, van Westrienen PE, Konings S, Nieboer ME, Veenhof C, Pisters MF (2021). Patients' perspectives on the usability of a blended approach to an integrated intervention for patients with medically unexplained physical symptoms: mixed methods study. J Med Internet Res.

[95] Fassier JB, Durand MJ, Caillard JF, Roquelaure Y, Loisel P (2015). Results of a feasibility study: barriers and facilitators in implementing the Sherbrooke model in France. Scand J Work Environ Health.

[96] Main CJ, Shaw WS, Nicholas MK, Linton SJ (2022). System-level efforts to address pain-related workplace challenges. Pain.

[97] Aschbrenner KA, Kruse G, Gallo JJ, Plano Clark VL (2022). Applying mixed methods to pilot feasibility studies to inform intervention trials. Pilot Feasibility Stud.

[98] Tewerkstelling per sector. Statistiek Vlaanderen.

[99] Svendsen MT, Bak CK, Sørensen K, Pelikan J, Riddersholm SJ, Skals RK, Mortensen RN, Maindal HT, Bøggild H, Nielsen G, Torp-Pedersen C (2020). Associations of health literacy with socioeconomic position, health risk behavior, and health status: a large national population-based survey among Danish adults. BMC Public Health.

[100] Arias López MD, Ong BA, Borrat Frigola X, Fernández AL, Hicklent RS, Obeles AJ, Rocimo AM, Celi LA (2023). Digital literacy as a new determinant of health: a scoping review. PLOS Digit Health.

[101] Sylvia LG, Bernstein EE, Hubbard JL, Keating L, Anderson EJ (2014). Practical guide to measuring physical activity. J Acad Nutr Diet.

