# Supplementary material for: Evaluating the Impact on Pain Perceptions, Pain Intensity, and Physical Activity of a Mobile App to Empower Employees With Musculoskeletal Pain: Mixed Methods Pilot Study
Source: JMIR Form Res. 2025 Jun 27;9:e67886. doi: 10.2196/67886 (PMC12254710; doi:10.2196/67886)
Supplement: Multimedia Appendix 1 [file formative_v9i1e67886_app1.docx]

**Multimedia Appendix 1.** Exploratory factor analysis of the Dutch Musculoskeletal Questionnaire.

|  | **Rotated factor loadings** | |
| --- | --- | --- |
| **Item “at work, how often do you…?”** | **Sedentary work** | **Physically demanding work** |
| (1) work standing for a prolonged time | -0.76^a^ | 0.56 |
| (2) work sitting for a prolonged time | 0.84^a^ | -0.49 |
| (3) work on a computer for a prolonged time | 0.77^a^ | -0.35 |
| (4) work kneeled or crouched for a prolonged time | -0.49 | 0.67^b^ |
| (5) move loads (more than 5 kg) | -0.28 | 0.93^b^ |
| (6) move heavy loads (more than 20 kg) | -0.28 | 0.96^b^ |
| (7) have to apply force with your arms or hands | NA | NA |
| (8) work with bumping or vibrating tools | -0.21 | 0.54 |
| (9) drive in vehicles | NA | NA |
| (10) work in uncomfortable positions | -0.42 | 0.61^b^ |
| (11) work in the same position for a prolonged time | 0.68 | -0.12 |
| (12) make the same movement with your arms/hands | NA | NA |

*Note. Item 7 was excluded from the analysis due to a technical issue in the online questionnaire. Items 9 and 12 were also removed because of poor correlations with other items (polychoric correlations < 0.3). Two factors with eigenvalues greater than 1.00 were retained, jointly explaining 80.49% of the variance. Factor loadings of 0.40 or higher were used to determine clustering. Factor 1, representing sedentary work, initially contained four items. However, removing item 11 improved internal consistency, resulting in a final Cronbach’s α of 0.89. Factor 2, representing physically demanding work, initially contained five items. Removing item 8 similarly improved internal consistency, resulting in a final Cronbach’s α of 0.86. Superscripts indicate item groupings: ‘a’ denotes items forming the sedentary work scale, and ‘b’ denotes items forming the physically demanding work scale. NA, not applicable.*
