# Supplementary material for: Evaluating the Impact on Pain Perceptions, Pain Intensity, and Physical Activity of a Mobile App to Empower Employees With Musculoskeletal Pain: Mixed Methods Pilot Study
Source: JMIR Form Res. 2025 Jun 27;9:e67886. doi: 10.2196/67886 (PMC12254710; doi:10.2196/67886)
Supplement: Multimedia Appendix 2 [file formative_v9i1e67886_app2.docx]

**Multimedia Appendix 2.** Baseline comparison between study completers and drop-outs.

|  | **Completed** | **Dropped out** |
| --- | --- | --- |
| **Variable** | **N = 19** | **N = 47** |
| Age (years), mean (SD) | 44 (12.0) | 41 (10.0) |
| Gender (female), n (%) | 16 (84.2) | 41 (87.2) |
| Degree (bachelor or higher), n (%) | 17 (89.5) | 38 (80.9) |
| Profession (healthcare), n (%) | 18 (94.7) | 42 (89.4) |
| Tenure (≥ 10 years), n (%) | 14 (73.7) | 23 (48.3) |
| Work arrangement (fulltime), n (%) | 8 (42.1) | 27 (57.4) |
| Multisite pain (yes), n (%) | 18 (94.7) | 41 (87.2) |
| Pain duration (≥ 3 months), n (%) | 18 (94.7) | 43 (91.5) |
| Pain-related sick leave (≥ 1 month), n (%) | 0 (0.0) | 3 (6.4) |
| Work-related factors |  |  |
| Physical job demands, mean (SD) | 6.7 (3.0) | 6.0 (2.6) |
| Workload, mean (SD) | 9.9 (2.6) | 9.4 (2.5) |
| Job autonomy, mean (SD) | 12.0 (4.7) | 13.3 (3.4) |
| Social support, mean (SD) | 14.2 (3.6) | 14.3 (3.4) |
| Maladaptive pain perceptions |  |  |
| Pain catastrophizing, mean (SD) | 13.0 (6.9) | 13.4 (8.0) |
| Fear-avoidance beliefs (activities), mean (SD) | 7.4 (4.2)* | 10.1 (4.1)* |
| Fear-avoidance beliefs (work), mean (SD) | 17.3 (7.6) | 14.4 (7.2) |
| Pain intensity (VAS), mean (SD) | 33.8 (19.5) | 32.5 (18.1) |
| Physical activity (daily step count), mean (SD) | 9934 (3116.4) | 9407 (2341.7) |

*Note. *P<.05. Dropout was defined as not completing the final follow up questionnaire.*
