# Supplementary material for: Evaluating the Impact on Pain Perceptions, Pain Intensity, and Physical Activity of a Mobile App to Empower Employees With Musculoskeletal Pain: Mixed Methods Pilot Study
Source: JMIR Form Res. 2025 Jun 27;9:e67886. doi: 10.2196/67886 (PMC12254710; doi:10.2196/67886)
Supplement: Multimedia Appendix 3 [file formative_v9i1e67886_app3.docx]

**Multimedia Appendix 3.** Regression slopes of outcome variables.


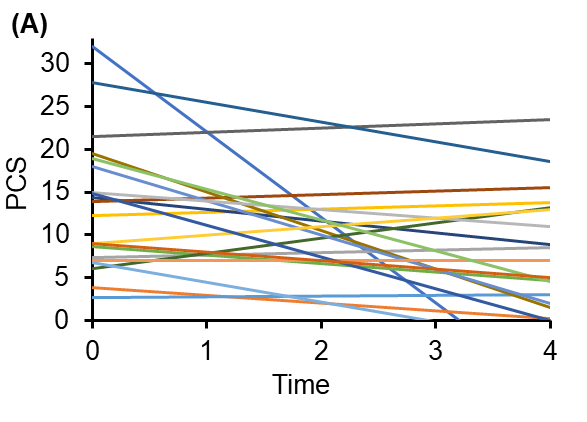

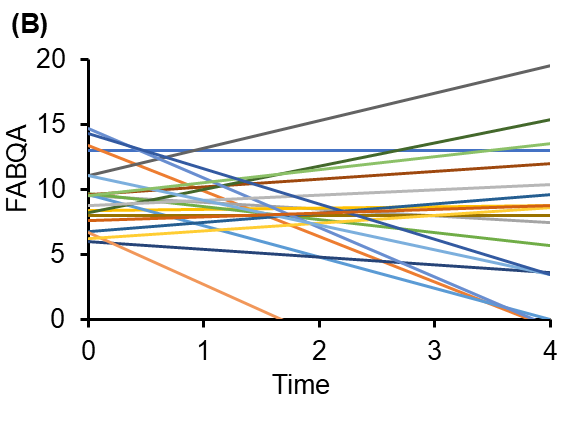


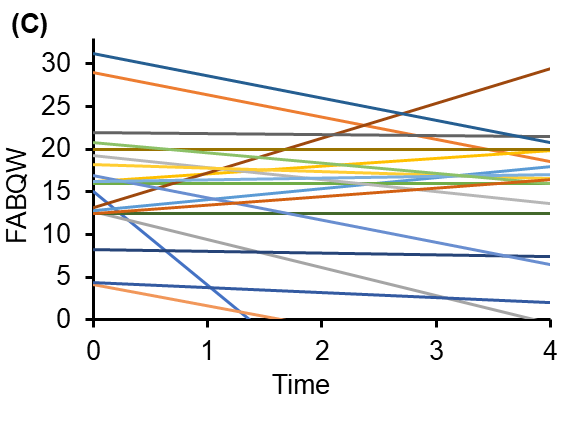

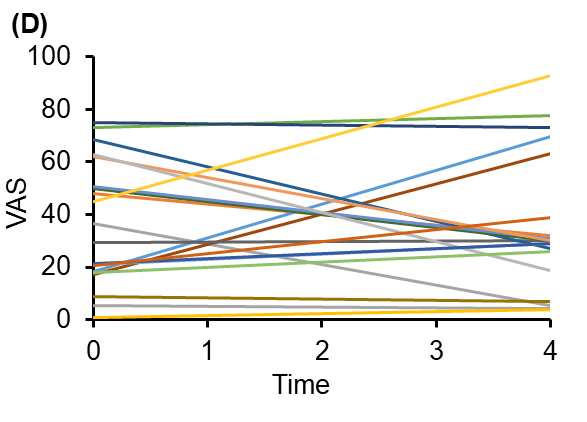


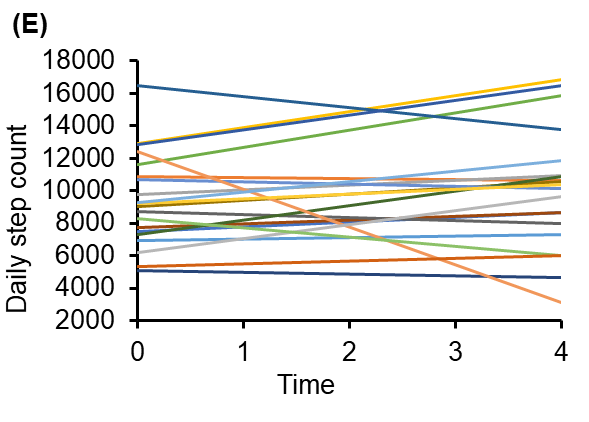


*Note. Regression slopes of 20 random participants are depicted. PCS, pain catastrophizing scale; FABQA, fear-avoidance beliefs questionnaire (physical activity subscale); FABQW, fear-avoidance beliefs questionnaire (work subscale); VAS, visual analogue scale*
